# Gnawing Between Cells and Cells in the Immune System: Friend or Foe? A Review of Trogocytosis

**DOI:** 10.3389/fimmu.2022.791006

**Published:** 2022-02-03

**Authors:** Siyu Zhao, Lichao Zhang, Suoyu Xiang, Yunyi Hu, Zhongdao Wu, Jia Shen

**Affiliations:** ^1^ Department of Parasitology of Zhongshan School of Medicine, Sun Yat-sen University, Guangzhou, China; ^2^ Key Laboratory of Tropical Disease Control (SYSU), Ministry of Education, Guangzhou, China; ^3^ Provincial Engineering Technology Research Center for Biological Vector Control, Guangzhou, China

**Keywords:** trogocytosis, information transmission, immune defense, immune escape, immune regulation

## Abstract

Trogocytosis occurs when one cell contacts and quickly nibbles another cell and is characterized by contact between living cells and rapid transfer of membrane fragments with functional integrity. Many immune cells are involved in this process, such as T cells, B cells, NK cells, APCs. The transferred membrane molecules including MHC molecules, costimulatory molecules, receptors, antigens, etc. An increasing number of studies have shown that trogocytosis plays an important role in the immune system and the occurrence of relevant diseases. Thus, whether trogocytosis is a friend or foe of the immune system is puzzling, and the precise mechanism underlying it has not yet been fully elucidated. Here, we provide an integrated view of the acquired findings on the connections between trogocytosis and the immune system.

## Trogocytosis

Trogocytosis, a form of cell-to-cell interaction widely existing in a species or between different species, involves one cell contacting and quickly “biting” another cell. This interaction was first described in 1970 as part of the process of parasites attacking and killing host cells (1). In 2002,it was given its name from the ancient Greek word “*trogo*”, which means “nibbling” to describe the phenomenon of the transfer of membrane fragments containing membrane-anchored proteins between cells (2).

Recently, an increasing number of studies have shown that trogocytosis plays a vital role in the immune system, including antigen presentation, T cell differentiation, immune regulation, and anti-infection and anti-tumor immunity (3–19). Many types of proteins are transferred between cells by trogocytosis, including MHC (major histocompatibility complex) molecules, costimulatory molecules, adhesion molecule receptors, tumor antigens, and the antigens of pathogens (9, 18, 20–27). The involved cell types include T cells (γδ T cells, and CD4^+^ and CD8^+^ αβ T cells), B cells, NK cells, dendritic cells, monocytes/macrophages, neutrophils, endothelial cells, fibroblasts, eosinophils, basophils), tumor cells, and pathogen cells (e.g., viruses, bacteria, and parasites) (9, 18, 20–28). Trogocytosis is strictly contact-dependent between living cells. Previous studies have shown that MHC I or HLA-C molecules on the surface of target cells of mice or humans are bidirectionally exchanged with the inhibitory receptor KIR (NK cell Ig-like receptor) of NK cells within a few minutes after coculture by immunological synapses (29, 30). In addition, Ralston et al. showed that amoebae only gnawed live-cell targets and directly engulfed dead cell corpses, which is one of the characteristics of trogocytosis (31). Interestingly, the membrane molecules obtained by trogocytosis, such as human leukocyte antigen (HLA)-C, peptide-MHC complexes and inhibitory natural killer cell receptors, can be recolonized on the surface of trogocytosis-positive cells without proteolytic cleavage and perform corresponding functions (29, 30, 32).

**Figure 1:**
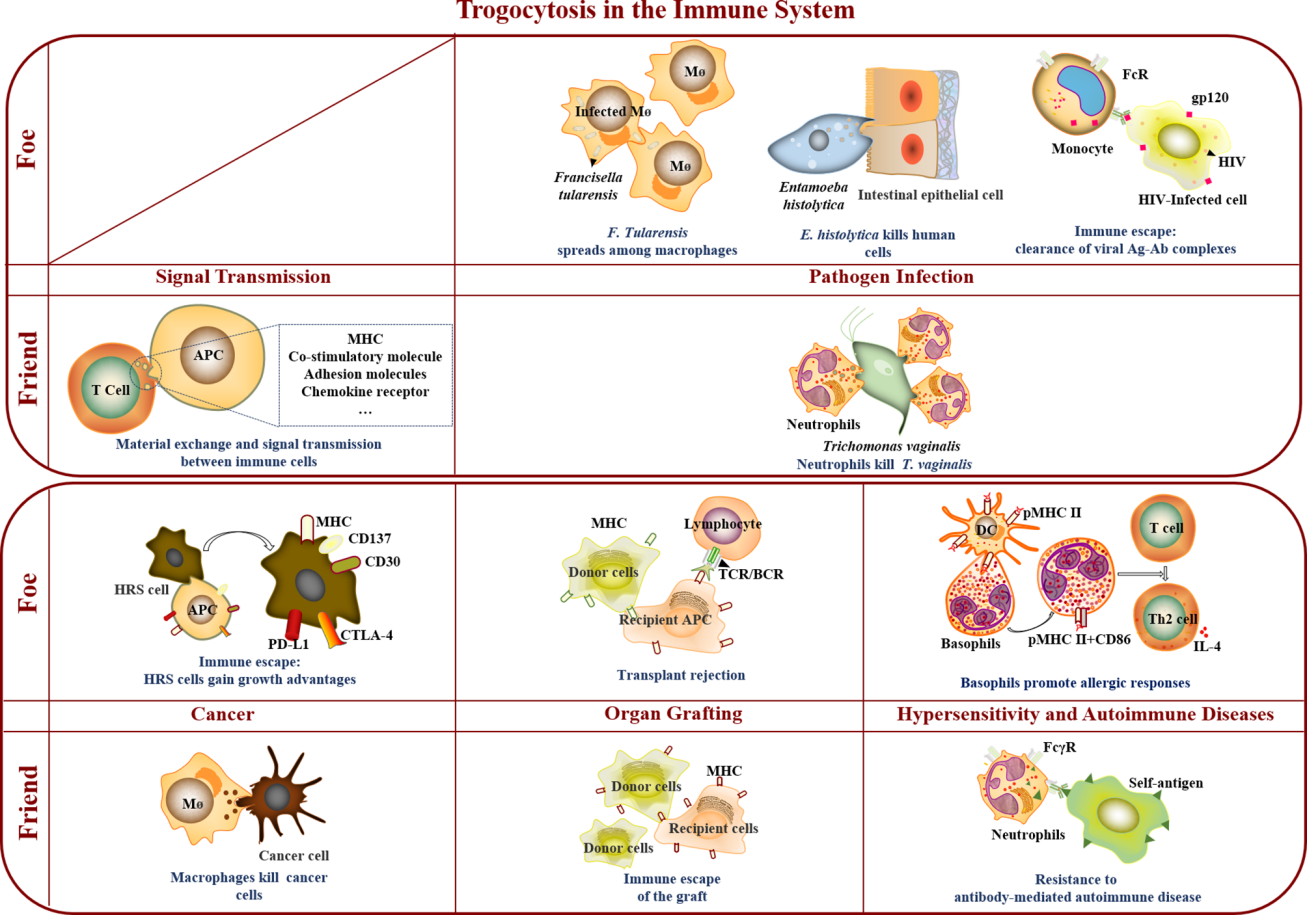
Trogocytosis in the immune system. In the immune system, trogocytosis mediates material exchange and signal transmission between immune cells, participates in immune cell killing of *T. vaginalis* and tumor cells, assists immune escape of grafts, and promotes resistance to antibody-mediated autoimmune disease. As a foe, trogocytosis accelerated *F. Tularensis* spreading among macrophages, and was involved in *E. histolytica* killing human cells, immune escape of virus and tumor cells, transplant rejection, and promotion of allergic responses.

The outcomes of trogocytosis, which vary with recipient cells, have changed our cognition of some classical theories. Trogocytosis does not always lead to the death of target cells. The interaction is relatively mild and tends to take up material and exchange information under physiological conditions, while it is often strong and usually ends in the death of target cells under pathological conditions (14, 20, 33). The existence of trogocytosis has shaken the traditional notion that cells can only perform their inherent functions or that a gene must be transcribed to use its protein product. With trogocytosis, even mature cells can perform a variety of unconventional functions, and these functions strictly depend on the cellular environment; that is, trogocytosis leads to very different functional consequences with similar processes in different states (34). However, is its function ultimately good or bad? From different perspectives, the role of trogocytosis also shifts between friend and foe in different environments. The reported physiological processes and related diseases involved in trogocytosis are summarized, divide into two aspects (i.e., friend and foe) and shown in **Figure 1**. Specifically, “Friend” refers to the biological events that trogocytosis is beneficial to appropriately enhancing human immunity and improving the ability to resist diseases, whereas “Foe” means the biological events that trogocytosis promotes the occurrence and development of diseases and harms human health.

## Trogocytosis in the Immune System

### The Immune System Transmits Signals Among Cells by Trogocytosis

The communication of immune cells involves various mechanisms, including immune synapses, nanotubes, trogocytosis, and exosomes. The transfer of membrane proteins of cells mediated by trogocytosis, including MHC molecules, CD3, costimulatory molecules, endothelial cell molecules, NK receptors, chemokine receptors, and glycosylphosphatidylinositol (GPI)-anchored proteins, widely occurs in T cells, B cells, macrophages, DC, NK cells. The interaction has modified the functions of immune cells involving in antigen presentation, information transfer, and regulation and homeostasis (3, 22, 25, 35–42).

In general, the typical manner for antigen presentation is that professional APCs (such as DCs) process the protein antigens into pMHCs (antigenic peptide-MHC complexes) and then present them to T cells. Some researches revealed a new antigen presentation pathway in which APCs and non-APCs directly obtained preformed pMHCs on the surface of target cells by trogocytosis and activated T cells without further processing (7, 10, 14, 19). Subsequently, the trogocytosis of MHCs occurs not only in T cell-APCs but also in T cell-endothelial cells, APC-APCs, APC-NK cells, tumor cell-T cells, and NK cells (43–47). For example, the transfer of pMHCs and CD80 from APCs to T cells could regulate T cell proliferative signals and sustain their activation in the absence of APCs (32, 48–50). At later time points, the number of activated CD4^+^ T cells trogocytosing and capturing MHC-peptide complexes increases and outnumbers the APCs, there is increasing probability that newly arriving T cells (and any T cells that have just been activated and need a second hit to continue to divide) would encounter them before encountering a proper professional APC. This T-T interaction leads to the inhibition of the newly arriving T cells and the Ag-experienced T cells, whereas naive T cells encountering antigen-presenting T cells are not inactivated, rather they are activated (51). In addition, Miyake et al. found that basophils gnawed DCs to obtain membrane fragments containing pMHC II and stimulated the proliferation of peptide-specific T cells, which indicated that trogocytosis could enhance the antigen presentation ability of basophils (52).

CD4^+^ T cells can obtain not only MHCs or pMHCs from APCs by trogocytosis but also costimulatory molecules (CD28, CD54, CD80) from APCs to deliver costimulatory signals to activate CD4^+^ T cells (48, 53, 54). Zhou et al. showed that MHC II and CD80 in CD4^+^ T cells maintained the activation of T cells in the absence of APC, which was an important factor in maintaining the homeostasis of memory T cells (50). Interestingly, the trogocytosed molecules might be unmodified in the recipient cell to perform their corresponding function. For example, Reed et al. reported that trogocytosis-mediated signaling has the potential to uniquely modulate the effector-cytokine production and differentiation of trog^+^ CD4^+^ T cell after separation from APC. To be more specific, trogocytosis-mediated intracellular signaling in CD4^+^ T cells drove Th2-associated effector cytokine production and differentiation (15). They found that trogocytosed molecules (pMHC complexes and costimulatory molecules) on trog^+^CD4^+^ T cells engaged their cognate receptors and drove the expression of IL-4 and GATA-3 in sequence, which was consistent with the differentiation of helper T cells type 2 (Th2) (6, 15, 16). Furthermore, extended trogocytosis-mediated signaling in CD4^+^ T cells resulted in the expression of Bcl-6, programmed cell death protein 1 (PD-1), CXCR5, and IL-21, which was consistent with the differentiation of follicular helper T cells (Tfhs) (5, 16). At the same time, IL-21 promoted the activation and survival of T cells and the generation of memory cells (55–59). On the other hand, trogocytosis might also affect the function of donor cell by removing some molecules from the donor cells. Qureshi et al. show that CTLA-4-expressing cells captured and removed CD80/CD86 from opposing cells to result in impaired costimulation *via* CD28, which revealed a mechanism of immune regulation whereby CTLA-4 acts as an effector molecule to inhibit CD28 costimulation by the cell-extrinsic depletion of ligands (60).

In addition to the transmission of costimulatory signals, trogocytosis also mediates other signals to be transmitted between different immune cells, which maintains immune homeostasis by balancing the activation and suppression of immune responses. It was recently reported that antigen-specific Treg cells form strong interactions with DCs to acquire DC-derived membranes by a process of trogocytosis, resulting in selective depletion of the complex of cognate pMHC II from the DC surface, reducing the capacity of DCs to present antigens (3). The strong binding of Tregs and their capacity to debilitate DC function in an antigen-specific manner, represents a novel pathway involving trogocytosis for Treg–mediated suppression and may be a mechanism by which Treg cells maintain immune homeostasis (3, 61). Trogocytosis also participates in the immunosuppressive effect mediated by Tregs in other ways. Tekguc et al. showed that Treg-expressed CTLA-4 depleted CD80/CD86 by trogocytosis and released free PD-L1 on APCs, which led to dual suppressive effects on T cell immune responses by limiting CD80/CD86 costimulatory naïve T cells and by increasing free PD-L1 available for the inhibition of effector T cells expressing PD-1 (17). Moreover, T cell microvilli-derived particles (TMPs) carrying T cell receptors (TCR) at all stages of T cell activation were separated from T cells by trogocytosis or membrane budding, which were deposited at the surface of cognate APCs to be a potentially effective pathway to transmit information on T cells to APCs (9).

It is reported that human CD8^+^ T cells played a regulatory role in the immune response by obtaining inhibitory molecules from APCs (62). Studies have shown that human CD8^+^ T cells obtained functional programmed death-ligand 1 (PD-L1) from APCs in an antigen-specific manner, which led to the apoptosis of neighboring T cells with the expression of the receptor of PD-1 (62). Gary et al. reported that the inhibitory molecule PD-L1 and the receptor PD-1 expressed on various human cells were transferred between immune cells by trogocytosis to regulate the immune response and recycle the molecule (62).

In addition to T cells, the antigen presentation and information transfer by trogocytosis also occurs in B cells. Soluble antigens can be bound to the B cell antigen receptor (BCR), and then internalized and presented to T cells by B cells, which initiates the humoral immune response. However, antigens are often insoluble or tethered to the cell surface. It has been reported that B cells can extract and present antigens that is tethered tightly to a noninternalizable surface and the evidence points to the major role being played by BCR-mediated wrenching of the antigen from its tether (63). Notably, a weak BCR can apparently wrench a tightly tethered antigen from the plate through continuous accumulation of effect in cases where the affinity difference is of several orders of magnitude (63). The nonstatic BCR antigen tether interaction and the motile nature of the cell may cause distortion of the antigen (and diminution of antigen tether affinity) (63). In addition, Xu et al. found that the BCR interacted with antigen-antibody complexes to remove epitopes from red blood cells by trogocytosis so that IgG could mediate the inhibitory effect on the immune response to antigens (27).

Furthermore, the trogocytosis also affects the functions of macrophages, DC, and NK cells. The trogocytosis by T cells is usually a process of the acquisition of antigens and signal transmission. In addition to the above functions, the trogocytosis by monocytes also involves a process of removing antigens, which show that the acquirer cell may control the functional outcome of trogocytosis (38). It was reported that the trogocytosis by macrophages mediated by FcγR affects the function of target cells, such as T cells and NK cells, but does not obtain new proteins or new functions (41). It is reported that KIR^+^ NK cells generally did not express CCR7; however, they were able to extract CCR7 from CCR7^+^ cells by trogocytosis to migrate to the site of killing mature DCs and T lymphoblasts with the help of chemokines CCL19/CCL21 (39, 40, 42).

Thus, the results showed that trogocytosis was widely involved in antigen presentation, information transfer, and regulation (activation, suppression, and even killing) of immune cells in the immune process, which was of greatly significance to the performance and homeostasis of immune function. Regrettably, the research on whether trogocytosed molecules undergoes degradation, modification and other processes before performing subsequent functions is so lacking that we know very little about this important issue. In addition, how cell membrane integrity is restored post trogocytosis is still a meaningful and not fully recognized process. In summary, our understanding of trogocytosis remains at a relatively superficial level and there are still many unknown fields waiting for us to explore, which also points out the direction for future research.

### Immune defense in Tumorigenesis and Pathogenic Microorganism Infection by Trogocytosis

According to the available literature, the trogocytosis by many immune cells, such as neutrophils, macrophages, and NK cells, played an essential role in immune defense in tumorigenesis and pathogen infection by destroying the membrane integrity of pathogens and tumor cells, which led to the loss or disability of organelles and the death of target cells (4, 11–13, 64).

#### Neutrophils Kill *Trichomonas vaginalis* by Trogocytosis

Previous studies have reported that the mechanisms of neutrophil clearance of *Trichomonas vaginalis* (*T. vaginalis*) included phagocytosis, toxic particles, and NETosis. Interestingly, Mercer et al. recently revealed that human neutrophils could successfully trogocytose *T. vaginalis* to achieve pathogen killing (12, 13). 3D and 4D live imaging showed that neutrophils rapidly surrounded and trogocytosed *T. vaginalis* before parasite death after coculture *in vivo*. *T. vaginalis* could only survive for approximately 8 minutes after trogocytosis started. During this process, the parasite experienced approximately 3 to 8 “bites” by an average of 3 to 6 neutrophils before parasite death. They also found that the trogocytosis by neutrophils and parasite killing depended on the presence of neutrophil serine protease and human serum factors, which reflected the synergistic effect of trogocytosis and toxic particles on parasite killing (12, 13). Furthermore, Leka et al. reported that complement receptor (CR) 3, which is known to bind iC3b, leading to phagocytosis, plays a role in mediating this trogocytosis of *T. vaginalis* (4). *T. vaginalis* is a unicellular parasite. When the membrane fragments removed from the parasite by trogocytosis are sufficient, or the membrane loss exceeds the repair capacity of *T. vaginalis*, the parasite will rupture and die. At this time, trogocytosis helps host to eliminate pathogens, which can be regarded as “Friend”. However, whether the trogocytosis by neutrophils or other immune cells can kill other multicellular pathogens, much larger than immune cells, merits future discussion.

#### Immune Cells Kill Tumor Cells by Trogocytosis

Recently, Matlung et al. reported that antibody-mediated neutrophil trogocytosis killed tumor cells in a contact-dependent manner, which did not release toxic particles and produced reactive oxygen species but only nibbled the plasma membrane of cancer cells, leading to a lytic type of cancer cell death. This mode of destruction of antibody-opsonized cancer cells by neutrophils, called trogoptosis, could be improved by inhibiting the CD47-SIRPα checkpoint (11). Similarly, Steele et al. showed that the continuous trogocytosis by macrophages could kill HER2-overexpressing breast cancer cells, which revealed that the trogocytosis mediated by macrophages led to the death of antibody-opsonized tumor cells (18). In addition, it was reported that human NK cells and T cells obtained the inner membrane protein H-RAS^G12V^ from tumor cells by trogocytosis, which induced the phosphorylation of extracellular regulated protein kinase ERK and promoted the secretion of INF-γ and TNF-α, the proliferation lymphocyte, and the efficiency of NK cells killing tumor cells (64). Moreover, Joshua et al. reported that when T cells acquire the surface molecules HLA-G and CD86 by trogocytosis, they differentiate into newly acquired Tregs to inhibit the escape of myeloma cells (8). The above example revealed that trogcytosis as a friend of host to suppress tumors.

In short, the emergence of trogocytosis by immune cells has deepened our understanding of antigen presentation, information transmission, and immune regulation and broadened the understanding of immune defense in the immune system. Trogocytosis, evidence of plasticity of the immune system, seems to have impacted traditional immunological theories, which suggests that immune cells can obtain functional molecules beyond their protein expression profile to perform functions independent of their main characteristics. For example, CD8^+^ T cells can obtain pMHC II, while CD4^+^ T cells can obtain pMHC I (48, 65). To some extent, the molecular expression profile on the surface of trogocytosis-positive cells has undergone subtle changes to temporarily display unconventional molecules. However, LeMaoult et al. suggested that APC-like T cells that arose *via* trogocytosis might not functionally compete with the professional APCs from which they took pMHCs and thus might not contribute to the immune response. Similarly, regulation of T-cell proliferative signals by acquired B7 molecules might be of more significant because it provides added value to an immune response by directly affecting the biology of the cell that has acquired CD80 (34). It was reported earlier that although trogocytosis might be an immune response booster from a quantitative standpoint, it should not induce qualitative changes (i.e., changes in the repertoire or function of the antigen-selected cells) and should not have a dramatic impact on immune responses (34). However, according to the previous studies, we can find that trogocytosis has extensively and deeply affected many aspects of the immune system, improving or reshaping people’s cognition of antigen presentation, information transmission, immune regulation, and immune defense.

## Trogocytosis in Diseases

### Immune Escape

Trogocytosis plays a bidirectional role in the immune system. On the one hand, the immune system uses it to defend against tumors and kill pathogens. On the other hand, abnormal cells and pathogens also escape immune system surveillance with the help of trogocytosis. Therefore, trogocytosis plays the dual role of “foe” or “friend” to maintain the homeostasis of the host and pathogens through a long-term balance in immune defense and immune escape.

It is reported that trogocytosis was significantly associated with spread of intracellular pathogens in mice, suggesting that direct bacterial transfer frequently occurs by this process *in vivo (* 26). Intracellular pathogens need to enter host cells to replicate. Pathogens must continue invading new target cells to survive and reproduce. During this process, pathogens may be exposed to the extracellular environment containing antibodies, complements, and other inhibitory factors, limiting pathogen spread. However, many reports have revealed that trogocytosis accelerates the spread of intracellular bacteria. Steele et al. found live *Francisella tularensis* (*F. tularensis*) and *Salmonella enterica* (*S. enterica*) were transferred from infected macrophages to uninfected macrophages using trogocytosis with the donor and recipient cells remaining intact, and then *F. tularensis* acquired from infected cells were found within double-membrane vesicles partially composed from the donor cell plasma membrane (26, 66).

Trogocytosis also plays an important role in promoting virus infection. During H5N1 influenza virus infection, B cells obtained the avian flu receptor of α2,3 sialic acids from monocytes using trogocytosis to increase their susceptibility to H5N1 virus infection (67). From another perspective, H5N1 influenza virus might utilize trogocytosis to expand its cell tropism and spread to immune cells with the lack of avian flu receptor (67). During HIV infection, monocytes can remove well-exposed antibody-gp120 complexes from the surface of infected cells through antibody-mediated trogocytosis, thereby evading surveillance of the immune system (21). In addition, Richardson et al. found that the transfer of envelope proteins of HIV-infected cells might promote antigen presentation more efficiently and extensively, strengthening antiviral immunity (24). Furthermore, it was reported that the cellular prion protein [PrP(C)], a GPI-anchored protein, is transferred between cells by trogocytosis, which may play an important role in the pathogenesis of prion disease (37). Moreover, Wu et al. showed that the T cell costimulatory molecule CD137 expressed in activated T cells and NK cells, which was induced by the *lmp-1* gene of Epstein-Barr virus (EBV) and the gene *tax* of human T cell virus (HTLV-1), was transferred to APCs by trogocytosis to form the CD137-CD137L complex. Subsequently, the complex was internalized and degraded, which weakened the function of T cell costimulation mediated by CD137 and promoted the immune escape of the virus (68).

**Figure 2:**
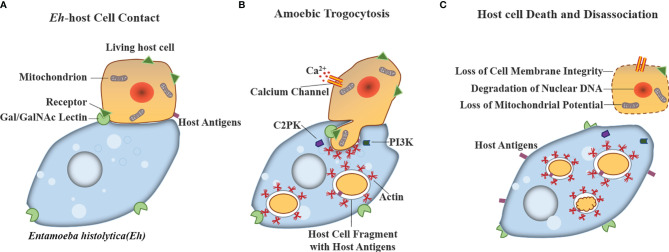
Model for host cell killing *via* amoebic trogocytosis. *Entamoeba histolytica* use trogocytosis to achieve kill host cells by three steps: **(A)** Contact to host glycoproteins containing Gal/GalNAc is mediated by the amoeba surface Gal/GalNAc lectin. **(B)** Amoebic trogocytosis is initiated. The process involves the signal transduction including PI3K and EhC2PK, both of which induce actin polymerization to ingest fragments of host cell material. Host cell intracellular calcium becomes elevated because of the activation of calcium channels. **(C)** Host cell death can be reflected in degradation of nuclear DNA and the loss of membrane integrity and mitochondrial potential.

The trogocytosis by parasites was reported in unicellular parasites, such as amoebae and *Trypanosoma*, which was beneficial to avoiding attack by host immune defenses and promoting their invasion and killing of host cells (33, 69, 70). Ralston et al. found that *Entamoeba histolytica* (*E. histolytica*) trophozoites use trogocytosis to achieve immune escape and kill host cells by destroying and ingesting host cell membrane fragments to obtain host antigens, which is called amoebic trogocytosis (33). Interestingly, unlike the trogocytosis observed between immune cell-mediated information transmission, amoebic trogocytosis was lethal, which resulted in the loss of cell membrane integrity, degradation of nuclear DNA, and loss of mitochondrial potential of target cells as shown in **Figure 2** (31, 70). In addition, Mukherjee et al. reported that trogocytosis occurred between the epimastigotes of *Trypanosoma cruzi* (69).

Similarly, trogocytosis-mediated immune escape was also observed in tumor progression. Zeng et al. found that the ectopic CD137 expression on Hodgkin and Reed-Sternberg (HRS) cells of Hodgkin’s lymphoma (HL) induced by EB virus suppressed immune responses *via* trogocytosis. The ectopically expressed CD137 on HRS cell could bind CD137L and cause an internalization of CD137L on the HRS cells themselves as well as on surrounding APCs, resulting in decreased PBMC proliferation and IFNγ secretion (68, 71). In conclusion, ectopic CD137 expression on HRS cells not only dampened immune activation by reducing CD137L levels on APCs, but also transduced an activation signal into HRS cells leading to the secretion of IL-13 weakening a cellular anti-HL immune responses by deviating immunity toward a Th2 response (71). In addition, CD30 (TNFRSF8) on HRS cells (72), PD-1 (73), and CTLA-4 on T cells were transferred to immune cells or tumor cells by trogocytosis, giving HRS cells a growth advantage and promoting the immune escape of HL (71).

### Attenuating Therapeutic Efficacy in Tumor Therapies

Trogocytosis is also involved in attenuating therapeutic efficacy in tumor therapies. Some studies have revealed that FcγR-mediated trogocytosis by immune cells reduces the effects of a variety of monoclonal antibody (mAb)-based therapies (74–78). Daratumumab, a monoclonal antibody against CD38, was applied to treat multiple myeloma (MM) by inhibiting the growth of tumor cells expressing CD38. Krejcik et al. discovered that monocytes’ and granulocytes’ gnawing of myeloma cells resulted in the loss of cell membrane fragments containing the daratumumab-CD38 complex, which led to the occurrence of daratumumab resistance during the treatment of MM (78). Similarly, the occurrence of rituximab (RTX) resistance, an anti-CD20 monoclonal antibody widely used in the treatment of non-Hodgkin’s lymphoma (77), mantle cell lymphoma (75), and chronic lymphocytic leukemia (76), was attributed to trogocytosis of CD20 on the surface of malignant B cells, which affected the therapeutic effect. Moreover, it was reported that the transfer of P-glycoprotein (functioning as an efflux pump of chemotherapeutics) between cells by trogocytosis led to the multidrug resistance of tumors (74). In addition, Chimeric antigen receptors (CARs) are artificially synthesized receptors that specifically reprogram the functions of T cells and are an effective new treatment for hematologic malignancies (79). However, researchers have found that some tumor cells were antigen-negative or antigen-low condition with unknown mechanism different from complete and permanent antigen loss, which could not be killed by CAR T cells and become a hidden danger that leads to tumor recurrence (80–82). Interestingly, Hamieh et al. revealed that CARs provoked reversible antigen loss through trogocytosis, an active process in which the target antigen is transferred to T cells, thereby decreasing the target density on tumor cells and abating T cell activity by promoting fratricide T cell killing and T cell exhaustion (82).

### Hypersensitivity and Autoimmune Diseases

Trogocytosis breaks the immune balance of the body, leading to hypersensitivity reactions and autoimmune diseases, which undoubtedly reflects as a “foe” of trogocytosis endangering human health. Miyake et al. reported that the transfer of large amounts of pMHC II from DCs to basophils by trogocytosis played an essential role in mouse atopic dermatitis by improving antigen presentation and promoting hypersensitivity reactions (52). In addition, Rossi et al. showed that epratuzumab (humanized anti-CD22 antibody), currently in clinical trials for B-cell lymphomas and autoimmune diseases, induced the reduction of multiple B-cell antigen receptor-modulating proteins (CD19, CD21, and CD79b) on the surface of B cells *via* their trogocytosis to effector cells. The reduction of CD19 levels was implicative for the efficacy of epratuzumab in autoimmune diseases because elevated CD19 had been correlated with susceptibility to SLE in animal models as well as in patients. The results suggest that B cells’ modulation of key regulatory proteins by trogocytosis may be an important mechanism for immunotherapy with autoimmune diseases (83).

### Transplant Rejection and Anti-Rejection

Trogocytosis plays both friend and foe roles in hematopoietic stem cell transplantation. Previous studies reported that the transfer of intact MHC-I molecules from the surface of the recipient cell to the donor cell by trogocytosis, on the one hand, was beneficial to transplants to avoid surveillance of host immune cells, which reduced rejection in the recipient to achieve successful transplantation (84, 85). On the other hand, allograft rejection is initiated by recipient T cells recognizing donor MHC molecules displayed on graft leukocytes migrating to the host’s lymphoid organs (86). Extensive and bidirectional transfer of MHC II molecules between donor and recipient cells *in vivo* was a continual process that through the life of the donor graft, which might involve trogocytosis (87, 88). *In utero* hematopoietic cellular transplantation (IUHCT) has great promise for treating congenital diseases of cellular dysfunction, such as sickle cell disease and immunodeficiency disorders. However, repeated failures in clinical cases of IUHCT in immunodeficiency disease force the establishment of the fetal immune system and prenatal tolerance. It was reported that the surface of tolerant host NK cells during development displayed a low level of donor MHC, and the trogocytosis-mediated transfer of donor MHCs to the recipient was an intrinsic mechanism for the regulation of the development and maintenance of NK cell tolerance in prenatal chimeras (89). The existing studies on trogocytosis involved in transplantation rejection mainly focus on blood system transplantation, while it remains to be explored whether trogocytosis participates in transplantation rejection of parenchymal organs, such as blood vessels, the heart, and kidneys.

In conclusion, trogocytosis is widely involved in various physiological processes and plays a vital role in the immune system and relevant diseases. The results show that trogocytosis is both a friend and a foe of the immune system in different immune environments (**Figure 1**). Trogocytosis-mediated material exchange and signal transmission between immune cells participated in killing *T. vaginalis* and tumor cells, assisted the immune escape of grafts, and promoted resistance to antibody-mediated autoimmune disease. As a foe, trogocytosis accelerated *F. Tularensis* spread among macrophages, took part in *E. histolytica* killing human cells, promoted allergic responses by basophils, assisted immune escape of virus and tumor cells, and aggravated transplant rejection.

## Regulatory Mechanism of Trogocytosis

The characteristic of the mechanism underlying trogocytosis is a key question, although the mechanism of trogocytosis has not been fully demonstrated. Related studies have shown that it is similar to phagocytosis. Trogocytosis was generally a biological process between cells mediated by the formation of the immunological synapse or by the contact of ligands (adhesion molecules, chemokines, antibodies, complements, etc.) and receptors (14, 22, 90–93) with involvement of actin and PI3K (Phosphoinositide 3-kinase) (22, 52, 94). In specific cell types, TC21, RhoG, Src, Syk intracellular calcium and myosin light-chain kinase also played an important role (22, 52, 94). All of these proteins above are also involved in phagocytosis except TC21 (94). When the receptors of trogocytosis-associated cells recognize the corresponding ligands on the target cells, the trogocytosis-associated cells undergo an energy-consuming process, including actin cytoskeletal remodeling and signal transmission and then membrane scission and cell engulfment occur (92, 95, 96). The membrane fragments containing ligands of target cells are captured by trogocytosis-associated cells and then either presented on the surface of trogocytosis-associated cells or internalized, processed, and degraded. Trogocytosis by T, B and NK cells occurs with formation of the immunological synapse (97, 98). CTL acquire membrane fragments with antigenic peptides from target cells through engagement of the T cell receptor (99). Harshyne et al. showed that DC trogocytosis obtained MHC molecules through phagocytic receptors (e.g., scavenger receptors) (7). In addition, FcγR- and complement receptor-mediated trogocytosis by monocytes, macrophages, and neutrophils was mainly involved in killing pathogens and eliminating autoantigens and autoantibodies (39, 100, 101). Sjöström et al. showed that murine NK cells acquired MHC I from surrounding cells by the inhibitory receptor Ly49 family and displayed them at the cell surface. At the same time, the uptake of the inhibitory ligand H-2D^d^ by NK cells was also dependent on the Ly49A receptor, and blocking the H-2Dd/Ly49A interaction would inhibit the uptake of H-2Dd (102). Another potential mechanism for trogocytosis is the transfer of APC molecules to the T cell *via* microclusters. Some researchers have observed small clusters of MHC being transferred from APC to T cells during the immune synapse (32, 97). Further support for microclusters as a mechanism for trogocytosis, is that microcluster formation is found to be resistant to treatment with PP2 (103).

The results of Aucher et al. showed that the trogocytosis by CD8^+^ and CD4^+^ T cells was partially or completely inhibited by inhibitors of cell activation, such as actin polymerization, kinase (such as Src-kinase, Syk-kinase, and PI3K), and low temperature (4°C), while B cells were not, which indicated that the trogocytosis by B cells might not depend on the process of cell activation. This difference was not attributed to the affinity of the B-cell receptor for its cognate antigen being higher than the affinity of the T-cell receptor for its antigen. Rather, it was related to the ability of trogocytosis-associated cells to conjugate with target cells in the presence of inhibitors (92). In addition, it was reported that amoebic trogocytosis could be inhibited by treatment with cytochalasin D, Gal/GalNAc (D-galactose/N-acetyl-D-galactosamine) lectin inhibitor, PI3K inhibitor, amoebic AGC kinase 1 (EhAGCK1) inhibitor or the mutation (96), amoebic cysteine ​​protease (EhCP) inhibitor (104), amoebic C2 domain protein kinase (EhC2PK) inhibitor, or mutation at low temperature (4°C), which led to a reduction in the killing rate of host cells (31). Among them, the AGC kinase family affects actin dynamics by manipulating the downstream PI3K, which influences the action of trogocytosis (**Figure 2**) (33, 105, 106). EhAGCK1 specifically participated in trogocytosis but not phagocytosis of dead cells, while EhAGCK2 participated in all actin-dependent endocytosis processes (96). Inhibition of EhCP could only block amoebic phagocytosis and could not block amoebic phagocytosis (104). The process of trogocytosis requires the participation of physiological temperature, actin rearrangement, Gal/GalNAc lectins, related enzymes (such as Src-kinase, Syk-kinase, and PI3K), and PI3K signals.

Pham et al. showed that the interruption of actin polymerization and lack of energy would block the process of trogocytosis, and blocking PI3K activity delayed the process of trogocytosis. Alternatively, the inhibition of Src kinases activity slowed the process and reduced the degree of trogocytosis (41). Previous studies have shown that inhibitors of trogocytosis mainly include ATPase inhibitors, actin skeleton blockers, Src, Syk, PIK3 pathway kinase inhibitors, and acidification inhibitors (62, 92, 107). Concanamycin A (an ATPase inhibitor) abrogated Ag-specific trogocytosis (62). Cytochalasin D (actin skeleton blocker) promoted actin depolymerization (41). Wortmannin (PI3K inhibitor) and PP2 (tyrosine-protein kinase Src inhibitor) significantly inhibited the trogocytosis by CD4^+^ and CD8^+^ T cells (41, 92). Picetanannol (a tyrosine-protein kinase Syk inhibitor) inhibited the trogocytosis by T cells and neutrophils (4). Ammonium chloride (an acidification inhibitor) reduced amoebic trogocytosis and cell killing but did not weaken initiation while inhibiting the process of receptor dependence (107).

Although the difference of mechanism between trogocytosis and phagocytosis is not yet clear, we have listed some aspects. Trogocytosis requires proteins involved in membrane bending and scission (95, 108) and a small GTPase (94), none of which normally has the role in engulfment and internalization of target cells during phagocytosis. There are many cells being capable of both trogocytosis and phagocytosis, such as neutrophils, macrophages, E. histolytica. How does a cell decide to initiate trogocytosis or phagocytosis? Receptor-ligand interactions may play certain roles. TCR, BCR, KIR, Ly49A receptor, phagocytic receptors, CR3, FcγR, EphA2, EphB and Gal/GalNAc Lectin receptor have been reported to be involved in the trogocytosis of different types of cells (3, 11–13, 15, 21, 27, 32, 40, 48, 62, 66–68, 95, 101, 108). However, whether the receptor is necessary for the initiation of gnawing remains unclear. Further, trogocytosis is regulated by Src and Syk protein kinases and is dependent on ATP, PKC, Ca^2+^, and actin cytoskeleton. GTPases (TC21, RhoG) and PI3K are also regarded as key factors in trogocytosis and are necessary for the trogocytosis by T cells to obtain MHC molecules from APCs mediated by TCR (**Table 1**) (44, 90, 92, 94). In other words, changes in the cytoskeleton initiate trogocytosis; therefore, the factors that affect the movement of the cytoskeleton can regulate trogocytosis. Finally, the ending of the cell that has been trogocytosed is an almost unexplored field. Why does trogocytosis cause cell death only under certain circumstances? Are additional factors, such as toxins, needed to kill cells through trogocytosis? Since trogocytosis requires direct contact, toxins would need to be highly specific to the cell that is being trogocytosed. How does the trogocytosed cell die post trogocytosis? Is the death of the trogocytosed cell attributed to the activation of the cell death pathway, or the accumulation of physical damage? Conversely, when trogocytosed cells are not killed by trogocytosis, how do they retain cellular integrity? Are cellular repair pathways activated in trogocytosed cells? All the above questions are worthy of our in-depth discussion.

**Table 1:** Compared the trogocytosis by different cell types.

Cell type		Involved Biological Aspects	Involved Receptors	Mechanisms (involved molecules)	Transferred Membrane Molecules	Outcomes	References
**Immune Cell**	**T cell**	To capture APC molecules (pMHC, costimulatory molecules);	TCR	Actin, PI3K, Small GTPase (TC21, RhoG), Src, Syk intracellular calcium, myosin light-chain kinase…	pMHC, Costimulatory molecules (CD28, CD54, CD80/CD86), RAS^G12V^	Regulating T cell proliferative signals and sustaining their activation; Inhibition of the newly arriving T cells and the Ag-experienced T cells; Modulating the effector-cytokine production, differentiation of trog^+^ CD4^+^ T cell and immune response; Promoting the efficiency of T and NK cells killing tumor cells	(5, 15, 16, 22, 32, 48–52, 55–60, 64, 94)
		To obtain the inner membrane protein H-RAS^G12V^ from tumor cells					
	**Treg cell**	To deplete pMHCII and CD80/CD86 from APCs;	TCR		pMHC II, CD80/CD86	Reducing the capacity of DCs to present antigen and maintain immune homeostasis; Suppressing T cell immune responses	(3, 17, 61)
	**CTL**	To acquire membrane fragments with antigenic peptides from target cells; To obtain inhibitory molecules (PD-L1) from APCs	TCR		PD-L1	Regulating the immune response and recycle the molecule	(62, 99)
**Immune Cell**	**B cell**	To extract and present antigen tethered tightly to a non-internalizable surface; To remove epitopes from red blood cells; To obtain the avian flu receptor of α2,3 sialic acids from monocytes	BCR	Actin, PI3K, , Small GTPase (TC21, RhoG), Src, Syk intracellular calcium, myosin light-chain kinase…	Antigen tethered tightly to a cell surface, Epitopes of red blood cells, Avian flu receptor (α2,3 sialic acids)	Initiating the humoral immune response; Mediating the inhibitory effect on the immune response; to increase their susceptibility to H5N1	(27, 63, 67)
	**NK**	To extract CCR7 from CCR7^+^ cells; To obtain the inner membrane protein H-RAS^G12V^ from tumor cells; To obtain and display MHC I	KIR, The inhibitory receptor Ly49 family (Ly49A receptor)		CCR7, H-RAS^G12V^, MHC I, H-2Dd	Migrating to the site of killing mature DCs and T lymphoblasts with the help of chemokines CCL19/CCL21; Promoting the secretion of INF-γ and TNF-α, the proliferation lymphocyte, and the efficiency of NK cells killing tumor cells; Preventing the NK cell-mediated killing of normal cells	(39, 40, 42, 64, 102)
	**DC**	To obtain MHC molecules	Phagocytic receptors (e.g., scavenger receptors)		MHC molecules, CD137	Antigen presentation	(7, 68)
**Immune Cell**	**Mø**	To remove antigens from target cells, such as T cells and NK cells; To participate in the death of antibody-opsonized tumor cells; Transfer of *F. tularensis* and *S. enterica* among macrophages	CR3	Actin, PI3K, Small GTPase (TC21, RhoG), Src, Syk intracellular calcium, myosin light-chain kinase…	Antigens, Intracellular pathogens (*F. tularensis* and *S. enterica*), CD137	Affecting the function of target cells but not obtain new proteins and new functions; Killing tumor cells; Accelerating the spread of intracellular bacteria	(18, 26, 38, 41, 66, 68)
			FcγR				
	**Neutrophil**	To nibble and remove membrane fragments from *Tv*; To participate in the destruction of antibody-opsonized cancer cells	CR3		Membrane fragments of *Tv* or cancer cell	Killing pathogens and tumor cells	(4, 11–13)
			FcγR				
	**Monocyte**	To remove well-exposed antibody-gp120 complexes from the surface of infected cells	CR3		Antibody-gp120 complexes	Resulting in immune escape of HIV; Killing pathogens	(21, 39, 100, 101)
			FcγR				
***E. histolytica***		To destroy and ingest host cell membrane fragments to obtain host antigens	Gal/GalNAc Lectin receptor	Actin, PI3K, Src, Syk, Gal/GalNAc lectin, EhAGCK1, EhAGCK2, EhCP, EhC2PK	Host antigens	Achieving immune escape and killing host cells	(31, 70)

T, T lymphocyte; B, B lymphocyte; APC, antigen presenting cell; Treg, regulatory T cell, CTL, cytotoxic lymphocyte; NK, natural killer cell; DC, dendritic cell; Mø, macrophage; pMHC, antigenic peptide-MHC complex; TCR, T cell receptor; BCR, B cell receptor; Tv, Trichomonas vaginalis. KIR, killer Ig-like receptors; CR3, complement receptor 3; FcγR, Fc gamma receptors; PI3K, phosphoinositide 3-kinase; Src, Src kinase family; Syk, Syk kinase family; HIV, human immunodeficiency virus; PGC, primordial germ cells; EphA2, Eph receptor A2; EphB, Eph receptor B; Gulp1, PTB domain-containing engulfment adapter protein 1; RacGEF, Rac-specific guanine nucleotide exchange factor; Tiam2, TIAM Rac1 Associated GEF 2; Gal/GalNAc, D-galactose/N-acetyl-D-galactosamine; EhAGCK1, amoebic AGC kinase 1; EhAGCK2, amoebic AGC kinase 2; EhCP, amoebic cysteine protease; EhC2PK, amoebic C2 domain protein kinase.

## Applications of Trogocytosis

As a profound demonstration of the function and characteristics of trogocytosis, potential applications of trogocytosis have been proposed. On the one hand, considering the adverse role of trogocytosis in the immune system, such as accelerating *F. Tularensis* spread among macrophages, participating in *E. histolytica* killing human cells, promoting allergic responses by basophils, assisting the immune escape of virus and tumor cells, and aggravating transplant rejection, the inhibition or disturbance of the process of trogocytosis is a potential treatment for some diseases. For example, as trogocytosis is strictly dependent on cell-to-cell contact, disturbing or destroying the process of contact may regulate or block subsequent events, which can provide new ideas for the treatment of diseases. Rossi et al. reported that the anti-CD22/CD20 bispecific antibody could be applied to treat lupus and other autoimmune diseases by reducing the depletion of B cells through enhancing trogocytosis (109). On the other hand, the characteristics and mechanism of trogocytosis can also be applied to many technical fields. Firstly, trogocytosis can be used in the auxiliary diagnosis. For example, due to the critical role of myelin-autoreactive T cells in peripheral blood in multiple sclerosis (MS), the detection of the recognition of T cells on their own APCs by protein transfer analysis can be used to assist in the diagnosis of MS, which is based on the ability of T cells to gnaw membrane proteins from autologous APCs (35). Secondly, trogocytosis can also be applied to find new TCR ligands. Generally, the discovery of TCR ligands has greatly facilitated the identification of disease-specific T cells. However, in many cases, specific ligands have not been defined because of the lack of an applicable method to detect new ligands. As a deep study of trogocytosis, Wang and colleagues used the phenomenon of trogocytosis to develop a cell-based selection platform to discover TCR ligands combined with a peptide-MHC library, which would help in studying the immune mechanisms of diseases and identify new targets for immunotherapies (110). In addition, Daubeuf and Puaux et al. proposed a method based on trogocytosis to capture APC membrane fragments combined with flow cytometry to detect, quantify, characterize and purify antigen-specific lymphocytes. The main advantage of this method is the compatible detection of the phenotypes and functional markers of lymphocytes, which were sorted and used in subsequent experiments or even treatment procedures (111, 112). Furthermore, it would be an exciting concept if trogocytosis could be used to engineer cell behavior, trafficking, and function. For example, CCR7 might be introduced on NK cells to alter their tissue recruitment based on the studies of Marcenaro and Somanchi (39, 42). In conclusion, the potential application of trogocytosis may involve other events and fields based on a deep understanding of the mechanism of trogocytosis in the future.

## Conclusion and Perspectives

In summary, trogocytosis plays a beneficial or adverse role in the immune system in different environments. On the one hand, trogocytosis strengthens immune defenses (antigen presentation and immune cell activation) and is accompanied by an increase in the probability of pathological immune damage. On the other hand, pathogens and abnormal cells also use trogocytosis to evade human immune surveillance. Based on existing research, it is impossible to generalize whether trogocytosis in the immune system is a foe or a friend because immunity is a double-edged sword. Trogocytosis can be applied to find therapeutic targets in infectious diseases, tumors and other diseases. However, given the negative role of trogocytosis in the immune system, disrupting its process of trogocytosis may be applied to treat the immune escape of tumors and pathogens, hypersensitivity, autoimmune diseases and graft-versus-host disease. At present, research on trogocytosis has not fully elucidated its mechanism and is mainly limited to the cytoskeleton’s role. Trogocytosis needs to be studied and clarified in depth.

There are several questions worth considering in future research. First, what are the different mechanisms by which trogocytosis regulates similar processes under different conditions, leading to different functional consequences? Second, because many cells are capable of trogocytosis and phagocytosis, such as *E. histolytica*, neutrophils, and macrophages, how does the cell decide to initiate trogocytosis or phagocytosis? Is it receptor-ligand binding or other unknown and more direct ways? Third, the proteins on the cell surface rely on hydrophobic interactions to exist stably on the cell membrane, so the transfer of proteins between cells needs to destroy or overcome this hydrophobic effect. The force required to pull out the intact membrane protein from the hydrophobic lipid bilayer as proven to be similar to the force required to break high-affinity protein interactions (e.g., antigen-antibody interactions) as early as 1978 (113), so how do trogocytosis-positive cells produce such a huge pulling force to “tear” membrane fragments and membrane proteins from the target cell? Finally, the problem of cell membrane repair post trogocytosis is also crucial. Are cellular repair pathways activated in trogocytosed cells? Specifically, since calcium influx is a trigger for cell membrane damage repair (114), does calcium influx occur in trogocytosed cells? As far as the result of trogocytosis is concerned, the rearrangement and combination of functions of cells through trogocytosis makes cells functions more diverse. Of course, trogocytosis somewhat improves the efficiency of organisms and reduces costs.

## Author Contributions

JS advocated writing this review. SZ collected literature and wrote the manuscript. LZ, SX, and YH collected literature. ZW provided some suggestions for this review. JS and ZW reviewed, edited and approved its final version. All authors read and approved the final version of the manuscript.

## Funding

This work was supported by grants from the National Natural Science Foundation of China (Grant No. 81802036 and 81871682), the National Research and Development Plan of China (No. 2020YFC1200100 and No. 2016YFC1200500), the Natural Science Foundation of Guangdong Province of China (2020A1515010896), the China Postdoctoral Science Foundation (No. 2018M631027 and 2019T120770).

## Conflict of Interest

The authors declare that the research was conducted in the absence of any commercial or financial relationships that could be construed as a potential conflict of interest.

## Publisher’s Note

All claims expressed in this article are solely those of the authors and do not necessarily represent those of their affiliated organizations, or those of the publisher, the editors and the reviewers. Any product that may be evaluated in this article, or claim that may be made by its manufacturer, is not guaranteed or endorsed by the publisher.

## References

[1] Culbertson CG. Pathogenic Naegleria and Hartmannella (Acenthamoeba). Ann New York Acad Sci (1970) 174(2):1018–22. doi: 10.1111/j.1749-6632.1970.tb45623.x 5278125

[2] Joly E Hudrisier D. What is Trogocytosis and What Is Its Purpose? Nat Immunol (2003) 4(9):815. doi: 10.1038/ni0903-815 12942076

[3] Akkaya B Oya Y Akkaya M Al Souz J Holstein AH Kamenyeva O. Regulatory T Cells Mediate Specific Suppression by Depleting Peptide-MHC Class II From Dendritic Cells. Nat Immunol (2019) 20(2):218–31. doi: 10.1038/s41590-018-0280-2 PMC6402611 30643268

[4] Aljona L Jose M. Investigating the Role of CR3 in Trogocytosis of Trichomonas Vaginalis Cells by Neutrophil-Like Cells. Pomona: California State Polytech Univ (2020).

[5] Chevalier N Jarrossay D Ho E Avery DT Ma CS Yu D. CXCR5 Expressing Human Central Memory CD4 T Cells and Their Relevance for Humoral Immune Responses. J Immunol (2011) 186(10):5556–68. doi: 10.4049/jimmunol.1002828 21471443

[6] Cook KD Miller J. TCR-Dependent Translational Control of GATA-3 Enhances Th2 Differentiation. J Immunol (2010) 185(6):3209–16. doi: 10.4049/jimmunol.0902544 PMC3993005 20696860

[7] Harshyne LA Zimmer MI Watkins SC Barratt-Boyes SM. A Role for Class A Scavenger Receptor in Dendritic Cell Nibbling From Live Cells. J Immunol (2003) 170(5):2302–9. doi: 10.4049/jimmunol.170.5.2302 12594251

[8] Joshua D Suen H Brown R Bryant C Ho PJ Hart D. The T Cell in Myeloma. Clin Lymp Myeloma Leuk (2016) 16(10):537–42. doi: 10.1016/j.clml.2016.08.003 27601001

[9] Kim HR Mun Y Lee KS Park YJ Park JS Park JH. T Cell Microvilli Constitute Immunological Synaptosomes That Carry Messages to Antigen-Presenting Cells. Nat Commun (2018) 9(1):3630. doi: 10.1038/s41467-018-06090-8 30194420 PMC6128830

[10] Li L Kim S Herndon JM Goedegebuure P Belt BA Satpathy AT. Cross-Dressed CD8alpha+/CD103+ Dendritic Cells Prime CD8+ T Cells Following Vaccination. Proc Natl Acad Sci USA (2012) 109(31):12716–21. doi: 10.1073/pnas.1203468109 PMC3411977 22802630

[11] Matlung HL Babes L Zhao XW van Houdt M Treffers LW van Rees DJ. Neutrophils Kill Antibody-Opsonized Cancer Cells by Trogoptosis. Cell Rep (2018) 23(13):3946–59 e6. doi: 10.1016/j.celrep.2018.05.082 29949776

[12] Mercer F Johnson PJ. Trichomonas Vaginalis: Pathogenesis, Symbiont Interactions, and Host Cell Immune Responses. Trends Parasitol (2018) 34(8):683–93. doi: 10.1016/j.pt.2018.05.006 PMC11132421 30056833

[13] Mercer F Ng SH Brown TM Boatman G Johnson PJ. Neutrophils Kill the Parasite Trichomonas Vaginalis Using Trogocytosis. PloS Biol (2018) 16(2):e2003885. doi: 10.1371/journal.pbio.2003885 29408891 PMC5815619

[14] Miyake K Karasuyama H. The Role of Trogocytosis in the Modulation of Immune Cell Functions. Cells (2021) 10(5):1255. doi: 10.3390/cells10051255 34069602 PMC8161413

[15] Reed J Wetzel SA. Trogocytosis-Mediated Intracellular Signaling in CD4(+) T Cells Drives TH2-Associated Effector Cytokine Production and Differentiation. J Immunol (2019) 202(10):2873–87. doi: 10.4049/jimmunol.1801577 PMC6504583 30962293

[16] Reed SJ. Impacts of Trogocytosis-Mediated Intracellular Signaling on CD4+ T Cell Effector Cytokine Production and Differentiation. University of Montana: University of Montana (2019). 10.4049/jimmunol.1801577 PMC6504583 30962293

[17] Tekguc M Wing JB Osaki M Long J Sakaguchi S. Treg-Expressed CTLA-4 Depletes CD80/CD86 by Trogocytosis, Releasing Free PD-L1 on Antigen-Presenting Cells. Proc Natl Acad Sci USA (2021) 118(30):e2023739118. doi: 10.1073/pnas.2023739118 34301886 PMC8325248

[18] Velmurugan R Challa DK Ram S Ober RJ Ward ES. Macrophage-Mediated Trogocytosis Leads to Death of Antibody-Opsonized Tumor Cells. Mol Cancer Ther (2016) 15(8):1879–89. doi: 10.1158/1535-7163.MCT-15-0335 PMC4975628 27226489

[19] Wakim LM Bevan MJ. Cross-Dressed Dendritic Cells Drive Memory CD8+ T-Cell Activation After Viral Infection. Nature (2011) 471(7340):629–32. doi: 10.1038/nature09863 PMC3423191 21455179

[20] Brown K Fidanboylu M Wong W. Intercellular Exchange of Surface Molecules and Its Physiological Relevance. Arch Immunol Ther Exp (Warsz) (2010) 58(4):263–72. doi: 10.1007/s00005-010-0085-y 20508995

[21] Guan Y. The First Structure of HIV-1 Gp120 With CD4 and CCR5 Receptors. Cell Biosci (2019) 9:2. doi: 10.1186/s13578-018-0267-6 30622696 PMC6317214

[22] Hudrisier D Aucher A Puaux AL Bordier C Joly E. Capture of Target Cell Membrane Components *via* Trogocytosis Is Triggered by a Selected Set of Surface Molecules on T or B Cells. J Immunol (2007) 178(6):3637–47. doi: 10.4049/jimmunol.178.6.3637 17339461

[23] Lee ST Paraskevas F. Macrophage–T Cell Interactions. I. The Uptake by T Cells of Fc Receptors Released From Macrophages. Cell Immunol (1978) 40(1):141–53. doi: 10.1016/0008-8749(78)90322-2 308861

[24] Richardson SI Crowther C Mkhize NN Morris L. Measuring the Ability of HIV-Specific Antibodies to Mediate Trogocytosis. J Immunol Methods (2018) 463:71–83. doi: 10.1016/j.jim.2018.09.009 30240705

[25] Smyth LA Afzali B Tsang J Lombardi G Lechler RI. Intercellular Transfer of MHC and Immunological Molecules: Molecular Mechanisms and Biological Significance. Am J Transplant Off J Am Soc Transplant Am Soc Transplant Surgeons (2007) 7(6):1442–9. doi: 10.1111/j.1600-6143.2007.01816.x PMC3815510 17511673

[26] Steele S Radlinski L Taft-Benz S Brunton J Kawula TH. Trogocytosis-Associated Cell to Cell Spread of Intracellular Bacterial Pathogens. eLife (2016) 5:e10625. doi: 10.7554/eLife.10625 26802627 PMC4786427

[27] Xu H Heyman B. IgG-Mediated Suppression of Antibody Responses: Hiding or Snatching Epitopes? Scand J Immunol (2020) 92(4):e12921. doi: 10.1111/sji.12921 32594540

[28] Reed J Reichelt M Wetzel SA. Lymphocytes and Trogocytosis-Mediated Signaling. Cells (2021) 10(6):1478. doi: 10.3390/cells10061478 34204661 PMC8231098

[29] Carlin LM Eleme K McCann FE Davis DM. Intercellular Transfer and Supramolecular Organization of Human Leukocyte Antigen C at Inhibitory Natural Killer Cell Immune Synapses. J Exp Med (2001) 194(10):1507–17. doi: 10.1084/jem.194.10.1507 PMC2193674 11714757

[30] Vanherberghen B Andersson K Carlin LM Nolte-'t Hoen EN Williams GS Hoglund P. Human and Murine Inhibitory Natural Killer Cell Receptors Transfer From Natural Killer Cells to Target Cells. Proc Natl Acad Sci USA (2004) 101(48):16873–8. doi: 10.1073/pnas.0406240101 PMC534731 15550544

[31] Ralston KS Solga MD Mackey-Lawrence NM Somlata Bhattacharya A Petri WA Jr. Trogocytosis by Entamoeba Histolytica Contributes to Cell Killing and Tissue Invasion. Nature (2014) 508(7497):526–30. doi: 10.1038/nature13242 PMC4006097 24717428

[32] Huang JF Yang Y Sepulveda H Shi W Hwang I Peterson PA. TCR-Mediated Internalization of Peptide-MHC Complexes Acquired by T Cells. Sci (New York NY) (1999) 286(5441):952–4. doi: 10.1126/science.286.5441.952 10542149

[33] Ralston KS. Taking a Bite: Amoebic Trogocytosis in Entamoeba Histolytica and Beyond. Curr Opin Microbiol (2015) 28:26–35. doi: 10.1016/j.mib.2015.07.009 26277085 PMC4542054

[34] LeMaoult J Caumartin J Carosella ED. Exchanges of Membrane Patches (Trogocytosis) Split Theoretical and Actual Functions of Immune Cells. Hum Immunol (2007) 68(4):240–3. doi: 10.1016/j.humimm.2006.11.001 17400058

[35] Bahbouhi B Pettre S Berthelot L Garcia A Elong Ngono A Degauque N. T Cell Recognition of Self-Antigen Presenting Cells by Protein Transfer Assay Reveals a High Frequency of Anti-Myelin T Cells in Multiple Sclerosis. Brain (2010) 133(Pt 6):1622–36. doi: 10.1093/brain/awq074 20435630

[36] Nakayama M Hori A Toyoura S Yamaguchi SI. Shaping of T Cell Functions by Trogocytosis. Cells (2021) 10(5):1155. doi: 10.3390/cells10051155 34068819 PMC8151334

[37] Liu T Li R Pan T Liu D Petersen RB Wong BS. Intercellular Transfer of the Cellular Prion Protein. J Biol Chem (2002) 277(49):47671–8. doi: 10.1074/jbc.M207458200 12359724

[38] HoWangYin KY Alegre E Daouya M Favier B Carosella ED LeMaoult J. Different Functional Outcomes of Intercellular Membrane Transfers to Monocytes and T Cells. Cell Mol Life Sci (2010) 67(7):1133–45. doi: 10.1007/s00018-009-0239-4 PMC11115494 20238479

[39] Marcenaro E Cantoni C Pesce S Prato C Pende D Agaugue S. Uptake of CCR7 and Acquisition of Migratory Properties by Human KIR+ NK Cells Interacting With Monocyte-Derived DC or EBV Cell Lines: Regulation by KIR/HLA-Class I Interaction. Blood (2009) 114(19):4108–16. doi: 10.1182/blood-2009-05-222265 19749090

[40] Marcenaro E Pesce S Sivori S Carlomagno S Moretta L Moretta A. KIR2DS1-Dependent Acquisition of CCR7 and Migratory Properties by Human NK Cells Interacting With Allogeneic HLA-C2+ DCs or T-Cell Blasts. Blood (2013) 121(17):3396–401. doi: 10.1182/blood-2012-09-458752 23449637

[41] Pham T Mero P Booth JW. Dynamics of Macrophage Trogocytosis of Rituximab-Coated B Cells. PloS One (2011) 6(1):e14498. doi: 10.1371/journal.pone.0014498 21264210 PMC3022012

[42] Somanchi SS Somanchi A Cooper LJ Lee DA. Engineering Lymph Node Homing of *Ex Vivo*-Expanded Human Natural Killer Cells *via* Trogocytosis of the Chemokine Receptor CCR7. Blood (2012) 119(22):5164–72. doi: 10.1182/blood-2011-11-389924 PMC3418772 22498742

[43] Roda-Navarro P Reyburn HT. Intercellular Protein Transfer at the NK Cell Immune Synapse: Mechanisms and Physiological Significance. FASEB J (2007) 21(8):1636–46. doi: 10.1096/fj.06-7488rev 17314139

[44] Rechavi O Goldstein I Kloog Y. Intercellular Exchange of Proteins: The Immune Cell Habit of Sharing. FEBS Lett (2009) 583(11):1792–9. doi: 10.1016/j.febslet.2009.03.014 19289124

[45] Dhainaut M Moser M. Regulation of Immune Reactivity by Intercellular Transfer. Front Immunol (2014) 5:112. doi: 10.3389/fimmu.2014.00112 24734030 PMC3975099

[46] Davis DM. Intercellular Transfer of Cell-Surface Proteins Is Common and can Affect Many Stages of an Immune Response. Nat Rev Immunol (2007) 7(3):238–43. doi: 10.1038/nri2020 17290299

[47] Brezinschek RI Oppenheimer-Marks N Lipsky PE. Activated T Cells Acquire Endothelial Cell Surface Determinants During Transendothelial Migration. J Immunol (1999) 162(3):1677–84. 9973429

[48] Hwang I Huang JF Kishimoto H Brunmark A Peterson PA Jackson MR. T Cells Can Use Either T Cell Receptor or CD28 Receptors to Absorb and Internalize Cell Surface Molecules Derived From Antigen-Presenting Cells. J Exp Med (2000) 191(7):1137–48. doi: 10.1084/jem.191.7.1137 PMC2193171 10748232

[49] Chung B Stuge TB Murad JP Beilhack G Andersen E Armstrong BD. Antigen-Specific Inhibition of High-Avidity T Cell Target Lysis by Low-Avidity T Cells *via* Trogocytosis. Cell Rep (2014) 8(3):871–82. doi: 10.1016/j.celrep.2014.06.052 PMC4174572 25088413

[50] Zhou J Tagaya Y Tolouei-Semnani R Schlom J Sabzevari H. Physiological Relevance of Antigen Presentasome (APS), an Acquired MHC/costimulatory Complex, in the Sustained Activation of CD4+ T Cells in the Absence of APCs. Blood (2005) 105(8):3238–46. doi: 10.1182/blood-2004-08-3236 15637136

[51] Helft J Jacquet A Joncker NT Grandjean I Dorothée G Kissenpfennig A. Antigen-Specific T-T Interactions Regulate CD4 T-Cell Expansion. Blood (2008) 112(4):1249–58. doi: 10.1182/blood-2007-09-114389 PMC2515122 18539897

[52] Miyake K Shiozawa N Nagao T Yoshikawa S Yamanishi Y Karasuyama H. Trogocytosis of Peptide-MHC Class II Complexes From Dendritic Cells Confers Antigen-Presenting Ability on Basophils. Proc Natl Acad Sci USA (2017) 114(5):1111–6. doi: 10.1073/pnas.1615973114 PMC5293035 28096423

[53] Xiang J Huang H Liu Y. A New Dynamic Model of CD8+ T Effector Cell Responses *via* CD4+ T Helper-Antigen-Presenting Cells. J Immunol (2005) 174(12):7497–505. doi: 10.4049/jimmunol.174.12.7497 15944248

[54] Tatari-Calderone Z Semnani RT Nutman TB Schlom J Sabzevari H. Acquisition of CD80 by Human T Cells at Early Stages of Activation: Functional Involvement of CD80 Acquisition in T Cell to T Cell Interaction. J Immunol (2002) 169(11):6162–9. doi: 10.4049/jimmunol.169.11.6162 12444120

[55] Yuan YQ Yang YP Huang XP. IL-21 Is Required for CD4 Memory Formation in Response to Viral Infection. JCI Insight (2017) 2(7:e90652). doi: 10.1172/jci.insight.90652 28405614 PMC5374067

[56] Novy P Huang XP Leonard WJ Yang YP. Intrinsic IL-21 Signaling Is Critical for CD8 T Cell Survival and Memory Formation in Response to Vaccinia Viral Infection. J Immunol (2011) 186(5):2729–38. doi: 10.4049/jimmunol.1003009 PMC3059504 21257966

[57] Kaji T Hijikata A Ishige A Kitami T Watanabe T Ohara O. CD4 Memory T Cells Develop and Acquire Functional Competence by Sequential Cognate Interactions and Stepwise Gene Regulation. Int Immunol (2016) 28(6):267–82. doi: 10.1093/intimm/dxv071 PMC4885215 26714588

[58] Khattar M Miyahara Y Schroder PM Xie AN Chen WH Stepkowski SM. Interleukin-21 Is a Critical Regulator of CD4 and CD8 T Cell Survival During Priming Under Interleukin-2 Deprivation Conditions. PloS One (2014) 9(1):e85882. doi: 10.1371/journal.pone.0085882 24416451 PMC3887105

[59] Peluso I Fantini MC Fina D Caruso R Boirivant M MacDonald TT. IL-21 Counteracts the Regulatory T Cell-Mediated Suppression of Human CD4+ T Lymphocytes. J Immunol (2007) 178(2):732–9. doi: 10.4049/jimmunol.178.2.732 17202333

[60] Qureshi OS Zheng Y Nakamura K Attridge K Manzotti C Schmidt EM. Trans-Endocytosis of CD80 and CD86: A Molecular Basis for the Cell-Extrinsic Function of CTLA-4. Sci (New York NY) (2011) 332(6029):600–3. doi: 10.1126/science.1202947 PMC3198051 21474713

[61] Ahmed KA Munegowda MA Xie Y Xiang J. Intercellular Trogocytosis Plays an Important Role in Modulation of Immune Responses. Cell Mol Immunol (2008) 5(4):261–9. doi: 10.1038/cmi.2008.32 PMC4651296 18761813

[62] Gary R Voelkl S Palmisano R Ullrich E Bosch JJ Mackensen A. Antigen-Specific Transfer of Functional Programmed Death Ligand 1 From Human APCs Onto CD8+ T Cells *via* Trogocytosis. J Immunol (2012) 188(2):744–52. doi: 10.4049/jimmunol.1101412 22174448

[63] Batista FD Neuberger MS. B Cells Extract and Present Immobilized Antigen: Implications for Affinity Discrimination. EMBO J (2000) 19(4):513–20. doi: 10.1093/emboj/19.4.513 PMC305589 10675320

[64] Rechavi O Goldstein I Vernitsky H Rotblat B Kloog Y. Intercellular Transfer of Oncogenic H-Ras at the Immunological Synapse. PloS One (2007) 2(11):e1204. doi: 10.1371/journal.pone.0001204 18030338 PMC2065899

[65] Lorber MI Loken MR Stall AM Fitch FW. I-A Antigens on Cloned Alloreactive Murine T Lymphocytes are Acquired Passively. J Immunol (1982) 128(6):2798–803. 6804568

[66] Steele SP Chamberlain Z Park J Kawula TH. Francisella Tularensis Enters a Double Membraned Compartment Following Cell-Cell Transfer. eLife (2019) 8:e45252. doi: 10.7554/eLife.45252 31017571 PMC6499538

[67] Kongsomros S Thanunchai M Manopwisedjaroen S Na-Ek P Wang SF Taechalertpaisarn T. Trogocytosis With Monocytes Associated With Increased Alpha2,3 Sialic Acid Expression on B Cells During H5N1 Influenza Virus Infection. PloS One (2020) 15(9):e0239488. doi: 10.1371/journal.pone.0239488 32946496 PMC7500609

[68] Wu M Wong HY Lin JL Moliner A Schwarz H. Induction of CD137 Expression by Viral Genes Reduces T Cell Costimulation. J Cell Physiol (2019) 234(11):21076–88. doi: 10.1002/jcp.28710 31025383

[69] Mukherjee S Mukhopadhyay A Andriani G Machado FS Ashton AW Huang H. Trypanosoma Cruzi Invasion Is Associated With Trogocytosis. Microbes Infect (2015) 17(1):62–70. doi: 10.1016/j.micinf.2014.10.009 25448052 PMC4302017

[70] Gilmartin AA Petri WA Jr. Exploring the Mechanism of Amebic Trogocytosis: The Role of Amebic Lysosomes. Microb Cell (2017) 5(1):1–3. doi: 10.15698/mic2018.01.606 29354646 PMC5772035

[71] Zeng Q Schwarz H. The Role of Trogocytosis in Immune Surveillance of Hodgkin Lymphoma. Oncoimmunology (2020) 9(1):1781334. doi: 10.1080/2162402X.2020.1781334 32934884 PMC7466850

[72] Nakashima M Watanabe M Uchimaru K Horie R. Trogocytosis of Ligand-Receptor Complex and its Intracellular Transport in CD30 Signalling. Biol Cell (2018) 110(5):109–24. doi: 10.1111/boc.201800002 29431186

[73] Kawashima M Carreras J Higuchi H Kotaki R Hoshina T Okuyama K. PD-L1/L2 Protein Levels Rapidly Increase on Monocytes *via* Trogocytosis From Tumor Cells in Classical Hodgkin Lymphoma. Leukemia (2020) 34(9):2405–17. doi: 10.1038/s41375-020-0737-9 32089543

[74] Levchenko A Mehta BM Niu X Kang G Villafania L Way D. Intercellular Transfer of P-Glycoprotein Mediates Acquired Multidrug Resistance in Tumor Cells. Proc Natl Acad Sci USA (2005) 102(6):1933–8. doi: 10.1073/pnas.0401851102 PMC545583 15671173

[75] Beum PV Kennedy AD Williams ME Lindorfer MA Taylor RP. The Shaving Reaction: Rituximab/CD20 Complexes Are Removed From Mantle Cell Lymphoma and Chronic Lymphocytic Leukemia Cells by THP-1 Monocytes. J Immunol (2006) 176(4):2600–9. doi: 10.4049/jimmunol.176.4.2600 16456022

[76] Williams ME Densmore JJ Pawluczkowycz AW Beum PV Kennedy AD Lindorfer MA. Thrice-Weekly Low-Dose Rituximab Decreases CD20 Loss *via* Shaving and Promotes Enhanced Targeting in Chronic Lymphocytic Leukemia. J Immunol (2006) 177(10):7435–43. doi: 10.4049/jimmunol.177.10.7435 17082663

[77] Boross P Jansen JHM Pastula A van der Poel CE Leusen JHW. Both Activating and Inhibitory Fc Gamma Receptors Mediate Rituximab-Induced Trogocytosis of CD20 in Mice. Immunol Lett (2012) 143(1):44–52. doi: 10.1016/j.imlet.2012.01.004 22285696

[78] Krejcik J Frerichs KA Nijhof IS van Kessel B van Velzen JF Bloem AC. Monocytes and Granulocytes Reduce CD38 Expression Levels on Myeloma Cells in Patients Treated With Daratumumab. Clin Cancer Res (2017) 23(24):7498–511. doi: 10.1158/1078-0432.CCR-17-2027 PMC5732844 29025767

[79] Brudno JN Kochenderfer JN. Recent Advances in CAR T-Cell Toxicity: Mechanisms, Manifestations and Management. Blood Rev (2019) 34:45–55. doi: 10.1016/j.blre.2018.11.002 30528964 PMC6628697

[80] Gardner R Wu D Cherian S Fang M Hanafi LA Finney O. Acquisition of a CD19-Negative Myeloid Phenotype Allows Immune Escape of MLL-Rearranged B-ALL From CD19 CAR-T-Cell Therapy. Blood (2016) 127(20):2406–10. doi: 10.1182/blood-2015-08-665547 PMC4874221 26907630

[81] Orlando EJ Han X Tribouley C Wood PA Leary RJ Riester M. Genetic Mechanisms of Target Antigen Loss in CAR19 Therapy of Acute Lymphoblastic Leukemia. Nat Med (2018) 24(10):1504–6. doi: 10.1038/s41591-018-0146-z 30275569

[82] Hamieh M Dobrin A Cabriolu A van der Stegen SJC Giavridis T Mansilla-Soto J. CAR T Cell Trogocytosis and Cooperative Killing Regulate Tumour Antigen Escape. Nature (2019) 568(7750):112–6. doi: 10.1038/s41586-019-1054-1 PMC6707377 30918399

[83] Rossi EA Goldenberg DM Michel R Rossi DL Wallace DJ Chang CH. Trogocytosis of Multiple B-Cell Surface Markers by CD22 Targeting With Epratuzumab. Blood (2013) 122(17):3020–9. doi: 10.1182/blood-2012-12-473744 23821660

[84] Chow T Whiteley J Li M Rogers IM. The Transfer of Host MHC Class I Protein Protects Donor Cells From NK Cell and Macrophage-Mediated Rejection During Hematopoietic Stem Cell Transplantation and Engraftment in Mice. Stem Cells (2013) 31(10):2242–52. doi: 10.1002/stem.1458 23818226

[85] Rogers IM. Trogocytosis in Allogeneic Transplants: Donor Cells Take on the Recipients Identity. Chimerism (2013) 4(4):142–3. doi: 10.4161/chim.26648 PMC3921197 24121536

[86] Marino J Babiker-Mohamed MH Crosby-Bertorini P Paster JT LeGuern C Germana S. Donor Exosomes Rather Than Passenger Leukocytes Initiate Alloreactive T Cell Responses After Transplantation. Sci Immunol (2016) 1(1):aaf8759. doi: 10.1126/sciimmunol.aaf8759 27942611 PMC5142759

[87] Brown K Meader L Nowocin A Edwards LA Cheung LH Smith RA. A Novel *In Vivo* Model Using Immunotoxin in the Absence of P-Glycoprotein to Achieve Ultra Selective Depletion of Target Cells: Applications in Trogocytosis and Beyond. J Immunol Methods (2020) 483:112794. doi: 10.1016/j.jim.2020.112794 32428450

[88] Brown K Sacks SH Wong W. Extensive and Bidirectional Transfer of Major Histocompatibility Complex Class II Molecules Between Donor and Recipient Cells *In Vivo* Following Solid Organ Transplantation. FASEB J (2008) 22(11):3776–84. doi: 10.1096/fj.08-107441 18632850

[89] Alhajjat AM Strong BS Durkin ET Turner LE Wadhwani RK Midura EF. Trogocytosis as a Mechanistic Link Between Chimerism and Prenatal Tolerance. Chimerism (2013) 4(4):126–31. doi: 10.4161/chim.26666 PMC3921193 24121538

[90] Tabiasco J Espinosa E Hudrisier D Joly E Fournie JJ Vercellone A. Active Trans-Synaptic Capture of Membrane Fragments by Natural Killer Cells. Eur J Immunol (2002) 32(5):1502–8. doi: 10.1002/1521-4141(200205)32:5<1502::AID-IMMU1502>3.0.CO;2-Y 11981839

[91] Tabiasco J Vercellone A Meggetto F Hudrisier D Brousset P Fournie JJ. Acquisition of Viral Receptor by NK Cells Through Immunological Synapse. J Immunol (2003) 170(12):5993–8. doi: 10.4049/jimmunol.170.12.5993 12794126

[92] Aucher A Magdeleine E Joly E Hudrisier D. Capture of Plasma Membrane Fragments From Target Cells by Trogocytosis Requires Signaling in T Cells But Not in B Cells. Blood (2008) 111(12):5621–8. doi: 10.1182/blood-2008-01-134155 PMC2424158 18381976

[93] Li KJ Wu CH Shen CY Kuo YM Yu CL Hsieh SC. Membrane Transfer From Mononuclear Cells to Polymorphonuclear Neutrophils Transduces Cell Survival and Activation Signals in the Recipient Cells *via* Anti-Extrinsic Apoptotic and MAP Kinase Signaling Pathways. PloS One (2016) 11(6):e0156262. doi: 10.1371/journal.pone.0156262 27258015 PMC4892539

[94] Martinez-Martin N Fernandez-Arenas E Cemerski S Delgado P Turner M Heuser J. T Cell Receptor Internalization From the Immunological Synapse Is Mediated by TC21 and RhoG GTPase-Dependent Phagocytosis. Immunity (2011) 35(2):208–22. doi: 10.1016/j.immuni.2011.06.003 PMC4033310 21820331

[95] Gong JY Gaitanos TN Luu O Huang YY Gaitanos L Lindner J. Gulp1 Controls Eph/ephrin Trogocytosis and is Important for Cell Rearrangements During Development. J Cell Biol (2019) 218(10):3455–71. doi: 10.1083/jcb.201901032 PMC6781437 31409653

[96] Somlata Nakada-Tsukui K Nozaki T. AGC Family Kinase 1 Participates in Trogocytosis But Not in Phagocytosis in Entamoeba Histolytica. Nat Commun (2017) 8(1):101. doi: 10.1038/s41467-017-00199-y 28740237 PMC5524646

[97] Osborne DG Wetzel SA. Trogocytosis Results in Sustained Intracellular Signaling in CD4(+) T Cells. J Immunol (2012) 189(10):4728–39. doi: 10.4049/jimmunol.1201507 PMC9631952 23066151

[98] Wetzel SA McKeithan TW Parker DC. Peptide-Specific Intercellular Transfer of MHC Class II to CD4+ T Cells Directly From the Immunological Synapse Upon Cellular Dissociation. J Immunol (2005) 174(1):80–9. doi: 10.4049/jimmunol.174.1.80 15611230

[99] Hudrisier D Riond J Mazarguil H Gairin JE Joly E. Cutting Edge: CTLs Rapidly Capture Membrane Fragments From Target Cells in a TCR Signaling-Dependent Manner. J Immunol (2001) 166(6):3645–9. doi: 10.4049/jimmunol.166.6.3645 11238601

[100] Masuda S Iwasaki S Tomaru U Baba T Katsumata K Ishizu A. Possible Implication of Fc Gamma Receptor-Mediated Trogocytosis in Susceptibility to Systemic Autoimmune Disease. Clin Dev Immunol (2013) 2013:345745. doi: 10.1155/2013/345745 24093044 PMC3777198

[101] Taylor RP Lindorfer MA. Fcgamma-Receptor-Mediated Trogocytosis Impacts mAb-Based Therapies: Historical Precedence and Recent Developments. Blood (2015) 125(5):762–6. doi: 10.1182/blood-2014-10-569244 25498911

[102] Sjostrom A Eriksson M Cerboni C Johansson MH Sentman CL Karre K. Acquisition of External Major Histocompatibility Complex Class I Molecules by Natural Killer Cells Expressing Inhibitory Ly49 Receptors. J Exp Med (2001) 194(10):1519–30. doi: 10.1084/jem.194.10.1519 PMC2193673 11714758

[103] Campi G Varma R Dustin ML. Actin and Agonist MHC-Peptide Complex-Dependent T Cell Receptor Microclusters as Scaffolds for Signaling. J Exp Med (2005) 202(8):1031–6. doi: 10.1084/jem.20051182 PMC1373686 16216891

[104] Gilmartin AA Ralston KS Petri WA. Inhibition of Amebic Cysteine Proteases Blocks Amebic Trogocytosis But Not Phagocytosis. J Infect Dis (2020) 221(10):1734–9. doi: 10.1093/infdis/jiz671 PMC7184912 31999350

[105] Jimenez C Portela RA Mellado M Rodriguez-Frade JM Collard J Serrano A. Role of the PI3K Regulatory Subunit in the Control of Actin Organization and Cell Migration. J Cell Biol (2000) 151(2):249–62. doi: 10.1083/jcb.151.2.249 PMC2192656 11038173

[106] Enomoto A Murakami H Asai N Morone N Watanabe T Kawai K. Akt/PKB Regulates Actin Organization and Cell Motility *via* Girdin/APE. Dev Cell (2005) 9(3):389–402. doi: 10.1016/j.devcel.2005.08.001 16139227

[107] Gilmartin AA Ralston KS Petri WA Jr Inhibition of Amebic Lysosomal Acidification Blocks Amebic Trogocytosis and Cell Killing. mBio (2017) 8(4):e01187–17. doi: 10.1128/mBio.01187-17 PMC5574710 28851845

[108] Abdu Y Maniscalco C Heddleston JM Chew TL Nance J. Developmentally Programmed Germ Cell Remodelling by Endodermal Cell Cannibalism. Nat Cell Biol (2016) 18(12):1302–+. doi: 10.1038/ncb3439 PMC5129868 27842058

[109] Rossi EA Chang CH Goldenberg DM. Anti-CD22/CD20 Bispecific Antibody With Enhanced Trogocytosis for Treatment of Lupus. PloS One (2014) 9(5):e98315. doi: 10.1371/journal.pone.0098315 24841238 PMC4026529

[110] Li G Bethune MT Wong S Joglekar AV Leonard MT Wang JK. T Cell Antigen Discovery *via* Trogocytosis. Nat Methods (2019) 16(2):183–90. doi: 10.1038/s41592-018-0305-7 PMC6719556 30700903

[111] Daubeuf S Puaux AL Joly E Hudrisier D. A Simple Trogocytosis-Based Method to Detect, Quantify, Characterize and Purify Antigen-Specific Live Lymphocytes by Flow Cytometry, *via* Their Capture of Membrane Fragments From Antigen-Presenting Cells. Nat Protoc (2006) 1(6):2536–42. doi: 10.1038/nprot.2006.400 17406507

[112] Puaux AL Campanaud J Salles A Preville X Timmerman B Joly E. A Very Rapid and Simple Assay Based on Trogocytosis to Detect and Measure Specific T and B Cell Reactivity by Flow Cytometry. Eur J Immunol (2006) 36(3):779–88. doi: 10.1002/eji.200535407 16482513

[113] Bell GI. Models for the Specific Adhesion of Cells to Cells. Sci (New York NY) (1978) 200(4342):618–27. doi: 10.1126/science.347575 347575

[114] Andrews NW Corrotte M. Plasma Membrane Repair. Curr Biol (2018) 28(8):R392–R7. doi: 10.1016/j.cub.2017.12.034 29689221

